# Electrodeposition and characterization of nanostructured composite Ni–W alloys for hydrogen evolution in basic media†

**DOI:** 10.1039/d5ra03136b

**Published:** 2025-07-01

**Authors:** Raiedhah A. Alsaiari, Alaa A. Abd-Ellah, Zeinab M. Anwar, Salah S. Shata, Medhat M. Kamel, Nasser Y. Mostafa

**Affiliations:** a Department of Chemistry, Faculty of Science and Arts in Sharurah, Najran University Sharurah Saudi Arabia; b Department of Chemistry, Faculty of Science, Suez Canal University Ismailia 41522 Egypt n.mostafa@science.suez.edu.eg +20 1113343594; c Geology Department, Faculty of Science, Suez Canal University Ismailia 41522 Egypt

## Abstract

The development of efficient, low-cost, and stable electrocatalysts is essential for hydrogen production *via* water splitting, which is a key technology for sustainable energy. This study investigates electrodeposited nanostructured composite Ni–W alloy thin films with varying tungsten contents (up to 35.8 wt%) as electrocatalysts for the hydrogen evolution reaction (HER) in a 1 mol L^−1^ KOH solution. The physical and chemical properties of the electrocatalytic composite coatings were thoroughly characterized using XRD, SEM, EDX and XPS. XRD confirmed their nanocrystalline structure and the presence of Ni_17_W_3_, metallic W, and WO_3_ phases. Scanning electron microscopy revealed that increasing tungsten content resulted in smaller particle sizes (from 23.9 nm for pure Ni to 7.3 nm for the highest W-content alloy) and a distinctive wrinkled surface morphology. The electrocatalytic performance for HER was evaluated using linear sweep voltammetry (LSV), Tafel plots, chronopotentiometry, and electrochemical impedance spectroscopy (EIS). These electrochemical analyses consistently demonstrated the incorporation of tungsten significantly enhanced HER activity of the Ni–W, and coating with the highest W-content (35.8 wt%) shows the best performance, characterized by the highest exchange current density (0.644 mA cm^−2^) and the lowest Tafel slope (−168 mV dec^−1^). Chronopotentiometry of the best catalyst (Ni–6W) proved that it sustains activity after 250 cycles, highlighting superior performance at a current density of −50 mA cm^−2^, and EIS confirmed its suitability in the alkaline electrolyte. These results underscore the potential of nanostructured composite Ni–W alloys as promising low-cost electrocatalysts for efficient hydrogen production. This work confirms the general feasibility and potential of composite Ni–W alloys as good electrocatalysts for HER.

## Abstract 2

Ni–6W demonstrated promise as a durable and efficient HER catalyst in basic media.

## Graphical abstract

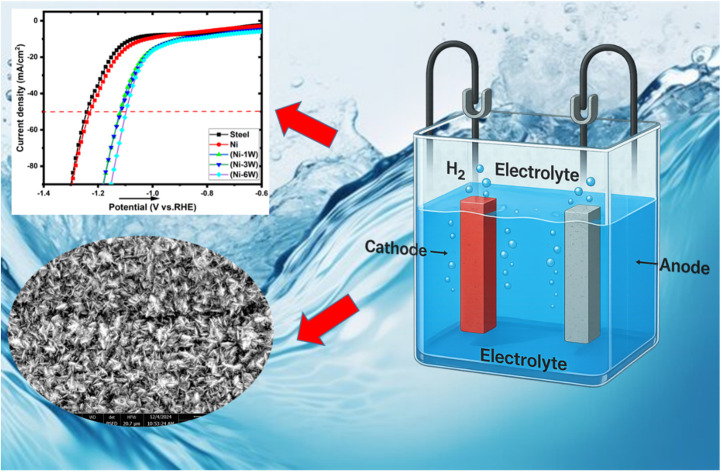

## 1. Introduction

As fossil fuel reserves dwindle and the demand for renewable energy grows, research into sustainable energy sources is crucial. Hydrogen is a clean energy carrier and a promising alternative to greenhouse gas-emitting fossil fuels.^1,2^ Beyond its potential as a fuel, hydrogen is also essential in industries like petroleum refining and ammonia production.^3–6^ This high global demand necessitates efficient, large-scale hydrogen production methods. While various methods exist, including steam reforming, coal gasification, and photoelectrochemical water splitting, steam reforming remains the dominant approach. However, this process is neither economical nor environmentally sound due to its high temperature requirements and significant CO_2_ emissions.^7^

Electrochemical water electrolysis, with its high energy efficiency, cleanliness, and ability to produce pure hydrogen, has recently been gained significant attention as a hydrogen production method. This process involves two reactions: one producing hydrogen at the cathode and the other producing oxygen at the anode. A major hurdle is the high energy consumption due to slow reaction rates, requiring a high overpotential (1.8–2.4 V), significantly more than the theoretical (1.23 V).^5^ While noble metals and their oxides are currently the best electrocatalysts, their scarcity and cost limit widespread industrial adoption, resulting in only about 4% of global hydrogen production coming from water electrolysis.^8^ Alkaline conditions are preferred for industrial hydrogen production due to the stability of transition metals and alloys (potential replacements for platinum and other noble metals).^9–12^ However, alkaline hydrogen evolution is less efficient and more energy-intensive than in acidic solutions due to higher overpotential requirements.^10,13,14^ Therefore, developing cost-effective, highly active, and long-lasting HER catalysts is essential for large-scale hydrogen production.

Many non-noble metal compounds, like transition metal phosphides,^15^ oxy-hydroxides,^16^ carbides,^17^ nitrides,^18^ selenides,^19^ sulfides,^20^ oxides,^21,22^ and alloys, are being actively researched as promising candidates for hydrogen production. These compounds have shown great potential for practical use. Among them, nickel-based alloys are particularly attractive due to their good electrocatalytic activity, stability, and the flexibility to create various compositions and morphologies, enhancing both intrinsic activity and active surface area. Electrodeposition is a particularly advantageous synthesis method for advanced coating.^23^ It allows for the creation of alloys with diverse morphologies, which can be further tailored by adjusting parameters like current density, applied potential, and deposition programs (*e.g.*, cyclic voltammetry or pulse deposition). Furthermore, electrodeposition is a binder-free method, avoiding increased interfacial resistance between the electrode and electrolyte, a problem often encountered with binder-containing methods. Finally, its mild operating conditions and low temperature make it an economic choice.^23,24^

Electrodeposited Ni–W alloys were widely studied as a replacement for hazardous hexavalent chromium coatings due to their superior wear and corrosion resistance.^25^ A study by Allahyarzadeh *et al.*^26^ has shown that Ni–W nanoparticles exhibit better HER activity and stability than pure Ni in acidic solutions, reducing the overpotential. In alkaline environments, Ni–W alloys are particularly promising for industrial hydrogen production because their excellent corrosion resistance translates to longer lifespans under harsh alkaline HER conditions.^13,27–31^ The HER activity of Ni–W alloys is strongly influenced by their composition, morphology, structure, and physicochemical properties, all of which can be controlled by adjusting the nickel and tungsten salt concentrations and electrodeposition parameters (pH, temperature, current density). Adding tungsten to the nickel lattice improves HER activity through a synergistic effect,^30^ while increased surface roughness provides more active sites.^28^ A study by Vernickaite *et al.*^13^ showed that among nickel, iron, and cobalt alloys with tungsten, the nickel–tungsten alloy demonstrated the best electrocatalytic properties for the HER and required the least energy to initiate the reaction. Their findings also indicated that higher tungsten content leads to the formation of nanostructured intermetallic compounds, which enhance HER performance.^13^

Jameeirad *et al.*^28^ produced a nickel–tungsten coating on a copper cathode using electroplating technique. They then improved the electrode's performance and HER activity by etching the surface to increase roughness and create a more active surface. Their work also explored the coating's wettability and its influence on hydrogen production.

A novel Ni–W–P/Mo catalyst for HER was synthesized *via* electrodeposition technique on a molybdenum mesh by Kong *et al.*^32^ The experimental results showed that when the current density was 10 mA cm^−2^, the best overpotential of Ni–W–P/Mo in alkaline medium was 75 mV. The Tafel slope (77 mV dec^−1^) also proved its excellent dynamic performance. In addition, the 60-hour stability test at a current density of 10 mA cm^−2^ indicated an excellent long-term stability performance. Porous and cracked Ni–W alloys with different W contents were prepared by Li *et al.*^33^ in a pyrophosphate bath. The results of electrochemical tests showed that the optimized coating is the PNiW10-6 catalyst, formed in the bath with a concentration of 100 g L^−1^ Na_2_WO_4_·2H_2_O at 0.6 A cm^−2^ for 5 min. The catalyst presented low overpotentials of 166 mV (*η*_10_), 213 mV (*η*_20_) and 269 mV (*η*_50_), high ECSA with 13 times the substrate Fe, high ECD of 0.741 mA cm^−2^ and low *R*_ct_ of 9.30 Ω cm^2^.

Ni–W alloy films were electrodeposited by de Paz-Castany and others^34^ from a gluconate aqueous bath at pH = 5.0, at varying current densities and temperatures. All films possessed ∼12 at% W. The kinetics of HER in 0.5 M H_2_SO_4_ indicated that the best-performing film was obtained at a current density of −4.8 mA cm^−2^ and 50 °C. Derakhshani *et al.*^35^ synthesized a nickel–tungsten coating and investigated its performance as a catalyst for the HER. They varied the current density during preparation and found that increasing it up to 500 mA cm^−2^ resulted in a columnar morphology. Electrochemical tests showed that higher plating current densities led to a five-fold increase in the active surface area. Derakhshani and others^36^ enhanced Ni–W coatings for HER by adding graphene oxide (GO), forming a porous, foam-like structure. They reported that adding 0.4 g L^−1^ GO significantly increased the electrode's active surface area and doubled its electrocatalytic properties.

This work investigates a new ecofriendly bath for creating nanostructured composite Ni–W alloy electrocatalysts for the HER. The method uses an alkaline bath containing lactate ions to electrodeposit Ni–W alloys of different compositions onto low-carbon steel cathode. The primary novelty of our work resides in the pioneering use of a lactate-based alkaline bath for the electrodeposition of nanostructured composite Ni–W alloys. This specific bath composition is distinct from previously reported methods and allows for the tailored synthesis of Ni–W coatings with tunable tungsten content and favorable surface morphologies (*e.g.*, wrinkled appearance), which directly correlate with enhanced HER performance in alkaline media.^26^ This approach differentiates our work from previous studies by leveraging an environmentally benign and economical ligand to achieve high-performance HER catalysts, providing a viable route for industrial application. The resulting electrocatalysts (Ni–W) were analyzed for their morphology, microstructure, and chemical composition using different techniques such as XRD, EDX, XPS, and SEM. Electrochemical methods, including linear sweep voltammetry, Tafel plots, chronopotentiometry, EIS, and electrochemical surface area (ECSA) measurements, were employed to evaluate the electrocatalytic activity, stability, and long-term performance of the deposited nanostructured composite Ni–W alloys.

## 2. Experimental details

### 2.1 Preparation of catalysts

Ni and composite Ni–W alloy coatings were electroplated onto low-carbon steel plates (3 cm × 3 cm × 0.05 cm) sourced from the Iron and Steel Company, Egypt. The steel plates' chemical composition is detailed in Table 1. Prior to electrodeposition, the steel plates underwent mechanical pretreatment involving sanding with progressively finer sandpaper to achieve a uniform, smooth surface. They were then degreased using a commercial degreaser and activated in a 20 vol% sulfuric acid solution at room temperature for one minute, followed by thorough rinsing with distilled water.^37^ A platinum plate (3 cm × 3 cm × 0.1 cm) from Alfa Aesar (Germany) served as the anode. The anode and cathode were connected to the positive and negative terminals of a power source, respectively.

**Table 1:** Chemical composition of steel substrate, wt%

C	Si	Mn	P	S	Al	Fe
0.08	0.01	0.03	0.025	0.025	0.045	The rest

Electroplating took place in a bath containing lactic acid, sodium sulfate, sodium tungstate, and nickel sulfate (Table 2), all supplied by Merck (Egypt). The bath temperature was maintained at 333 K, and the pH was adjusted to 8. The total metal concentration (Ni + W) in the plating bath was fixed at 0.1 mol L^−1^. A two-electrode cell, constructed from Perspex with a platinum anode and a steel cathode, was used for galvanostatic electrodeposition. After a 30-minute deposition period, the steel plates were removed, rinsed with distilled water, dried, and weighed. The composition of the deposited alloys was determined using an atomic absorption spectrometer (PerkinElmer PinAAcle 500) after dissolving the deposits in 50% nitric acid and diluting with double-distilled water.

**Table 2:** Bath composition and operating conditions of the electroplating process

Composition and operating conditions		Test samples			
		Ni	Ni–1W	Ni–3W	Ni–6W
Bath composition	NiSO_4_·7H_2_O (M)	0.09	0.09	0.07	0.04
	Na_2_WO_4_·2H_2_O (M)	—	0.01	0.03	0.06
	CH_3_CH(OH)COOH (M)	0.50	0.50	0.50	0.50
	Anhydrous Na_2_SO_4_ (M)	0.20	0.20	0.20	0.20
Operating conditions	pH	8	8	8	8
	Current density (mA cm^−2^)	10	10	10	10
	Deposition time (minutes)	30	30	30	30
	Temperature (K)	333	333	333	333

### 2.2 Electrocatalysts characterization

The surface morphology, structure, and elemental composition of the samples were investigated using SEM and EDX, specifically with a QUANTA FEG 250 SEM-EDX instrument. Crystal structures were determined through XRD using a Rigaku MiniFlex 600 diffractometer with Cu-K_α_ radiation (*λ* = 0.1540 nm). X-ray photoelectron spectroscopy (XPS, SPECS – Surface Nano Analysis GmbH Version 4.89.2-r104748, Germany) was employed to analyze the surface element valence distribution. A smart background was utilized in the XPS analysis, and peak fitting was conducted with a 30% Lorentzian/Gaussian mixed ratio. The crystalline size (*D*) was calculated *via* the Scherrer equation.^38^1
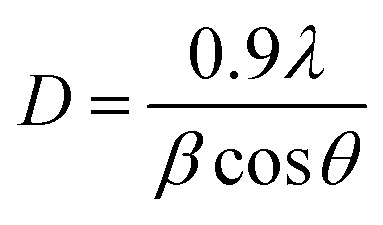
In this equation, *λ* stands for the wavelength of the X-rays, *β* represents the width of the diffraction peak at half its maximum height (FWHM), and *θ* is the angle at which the X-rays are reflected.

### 2.3 Electrochemical characterization

Electrocatalytic performance for the HER was evaluated by a standard three-electrode cell. A Pt electrode served as the auxiliary electrode, a saturated Ag/AgCl electrode in KCl solution was the reference electrode, and the coated samples functioned as the working electrode. A small area (0.03 cm^2^) of the alloy sample (Ni–1W, Ni–3W, and Ni–6W) was exposed to a 1 mol L^−1^ KOH solution at 298 K. LSV at a scan rate of 50 mV s^−1^ and EIS from 100 kHz to 0.1 Hz with a 5 mV amplitude were used to assess the HER electrocatalytic activity of the deposits. The EIS data was fitted using PS Trace software to determine the equivalent circuit.

The stability and durability of the electrocatalysts were evaluated by performing 250 LSV cycles with a 50 mV s^−1^ scan rate. Long-term stability was further investigated using chronopotentiometric analysis at a fixed current of −50 mA cm^−2^ for 72 hours. The ECSA values were investigated by cyclic voltammetry at scan rates ranging from 10 to 100 mV s^−1^.

## 3. Results and discussion

### 3.1 Electrodeposition of Ni–W alloy: a mechanistic study

Ni–W alloy plating falls under the “induced co-deposition” category according to Brenner's classification.^39^ Tungsten and molybdenum ions cannot be completely reduced in aqueous solutions on their own. However, they can be fully reduced and co-deposited as an alloy on the cathode surface with the addition of iron group metals.^39^ This phenomenon, where certain metals like tungsten and molybdenum are reduced alongside iron group metals (Fe, Ni, and Co), is termed induced co-deposition. Holt and Vaaler^40^ first proposed a theoretical framework to explain this process. The mechanism for tungsten reduction during co-deposition with nickel in lactate baths is like that observed with citrate ions.^41^Eqn (2)–(5) detail the proposed mechanism for Ni–W alloy electrodeposition using lactate anions (Lac^−^) as a ligand.

2 Ni^2+^ + Lac^−^ ↔ [Ni(Lac)]^+^

3 WO_4_^2−^ + Lac^−^ + H^+^ ↔ [(WO_4_)(Lac)(H)]^2−^

4 [Ni(Lac)]^+^ + [(WO_4_)(Lac)(H)]^2−^ ↔ [(Ni)(WO_4_)(Lac)(H)] + Lac^−^

5 [(Ni)(WO_4_)(Lac)(H)] + 3H_2_O + 8e^−^ ↔ Ni–W + Lac^−^ + 7OH^−^

Increasing current density during Ni–3W alloy electrodeposition leads to a higher tungsten percentage in the resulting coating (Table 3). This is because higher current densities favor tungsten's cathodic polarization and deposition. While nickel ions are preferentially deposited, the increased current density enhances the deposition rate of metals with more negative potentials, thus boosting the tungsten content.^42^

**Table 3:** Effect of current density on the composition of the Ni–3W alloy

Expt	Current density (mA cm^−2^)	Tungsten (wt%)
1	3.33	6.35
2	5.55	10.25
3	10.00	16.17

### 3.2 Characterization of electrocatalysts

#### 3.2.1 SEM study

**Fig. 1:**
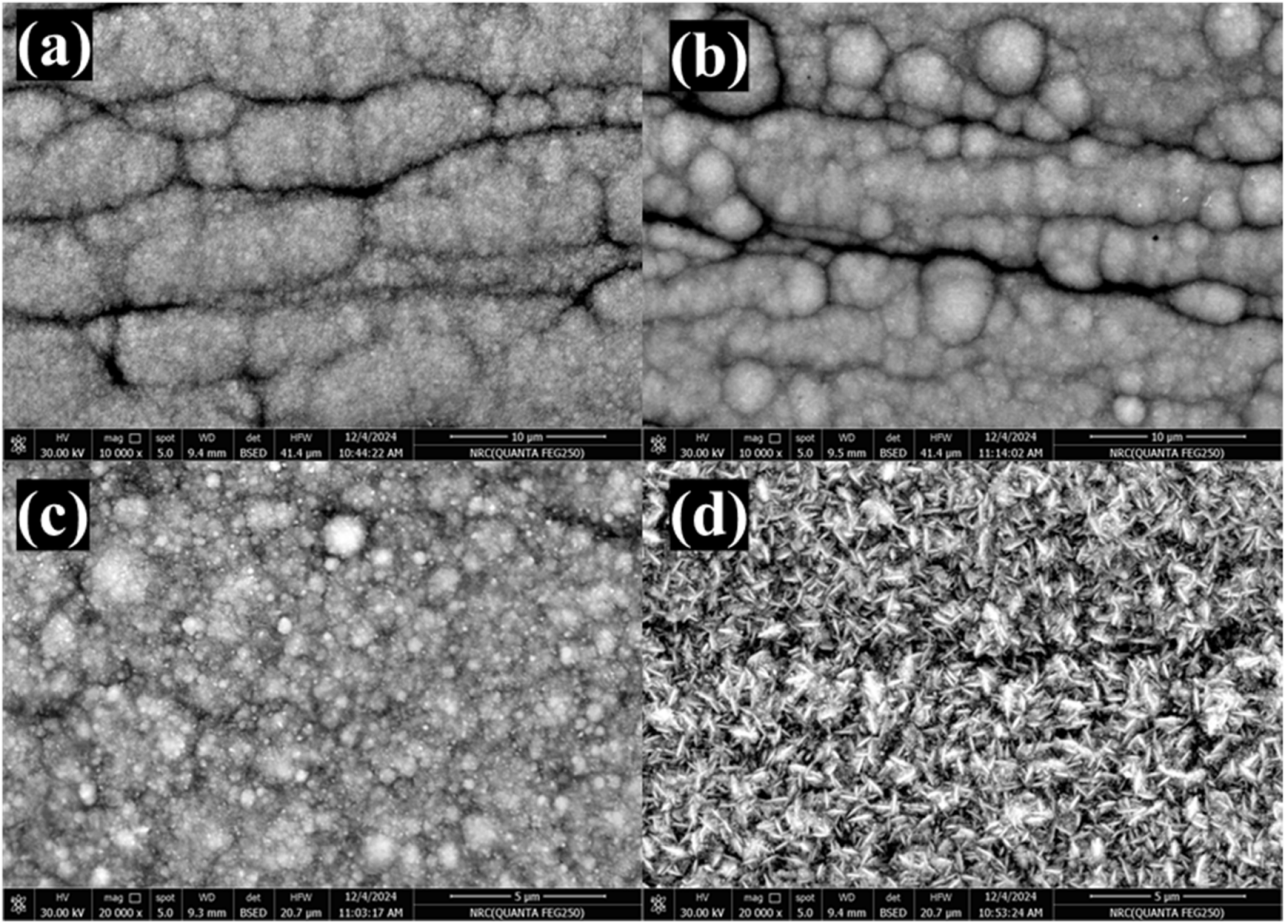
SEM micrographs of electrodeposited Ni (a), Ni–1W (b), Ni–3W (c), and Ni–6W (d) alloys.

Fig. 1 presents SEM micrographs illustrating the surface appearance of electrodeposited Ni and composite Ni–W alloy coatings on steel substrates. All coatings were observed to be homogeneous, compact, and well adhered to the substrate. The pure nickel coating (Fig. 1a) exhibited a typical columnar growth structure, with individual nickel columns oriented perpendicular to the substrate surface. Increasing the tungsten amount in composite Ni–W alloy coatings led to distinct changes in morphology. At low tungsten content of 10.21 wt% (Fig. 1b) and 16.17 wt% (Fig. 1c), the coatings appeared to have closely packed granular or fine nodular features, overlaid with an apparent network of cracks or fissures.^43^ Finally, at the highest tungsten content of 35.77 wt% (Fig. 1d), the coating displayed a unique cracked and wrinkled appearance, suggesting a change in the growth mechanism and/or stress state compared to the lower tungsten content alloys. This structure is known to enhance the availability of active areas for the HER.^37^ This progression in morphology with increasing tungsten content highlights the influence of alloy composition on the electrodeposition process and the resulting microstructure.

#### 3.2.2 EDX analyses

**Fig. 2:**
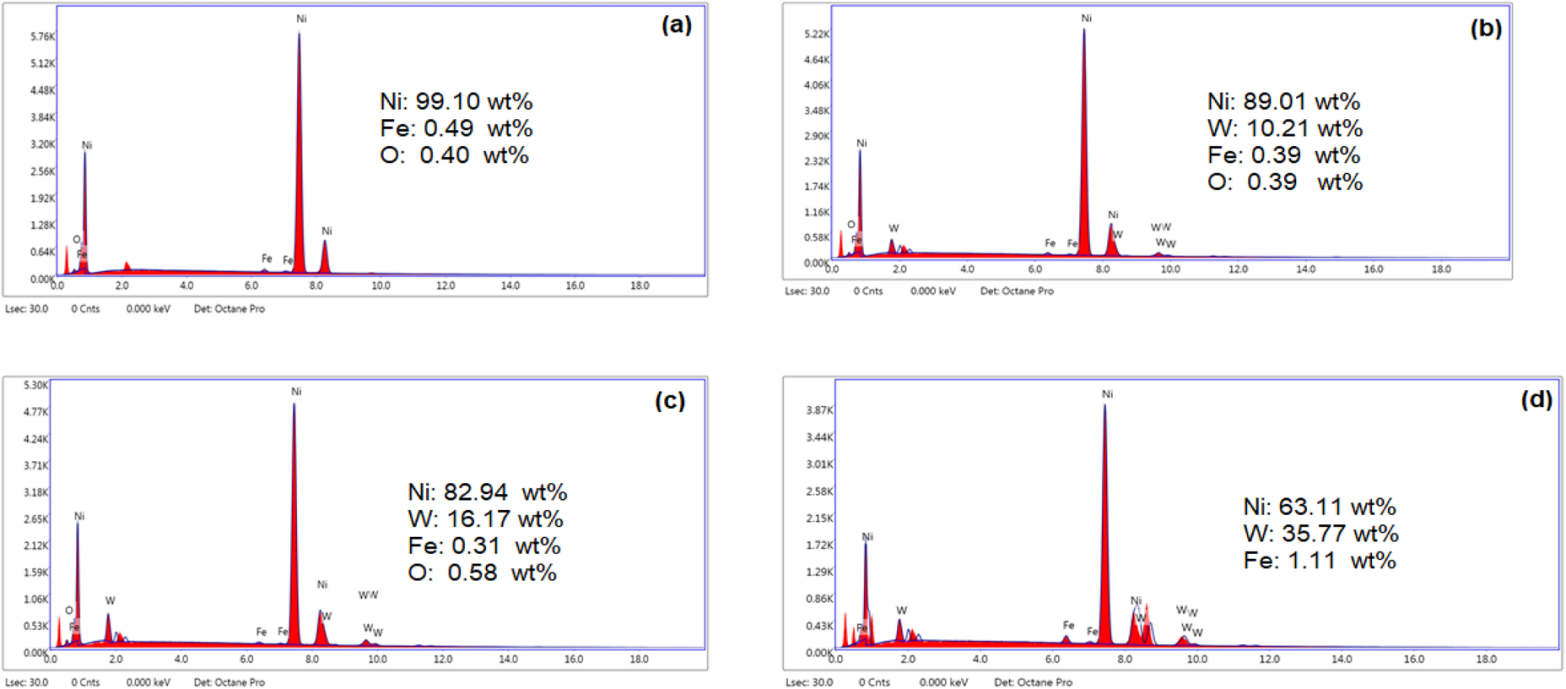
EDX spectra of electrodeposited Ni (a), Ni–1W (b), Ni–3W (c), and Ni–6W (d) alloys.

Fig. 2 shows the compositional EDX analysis of the Ni and composite Ni–W alloy coatings, which were electroplated from a lactate bath. The spectrum for the pure nickel coating (Fig. 2a) confirms that it is primarily composed of nickel. The presence of a small iron (Fe) peak indicates that the nickel coating is thin, as the electron beam penetrates to the steel substrate. The EDX spectra for all composite Ni–W alloy coatings (Fig. 2b–d) show characteristic peaks for both Ni and W, confirming that both elements were deposited together.^44^ The amount of tungsten in the coatings increases with increasing concentration of sodium tungstate in the plating bath. Specifically, the tungsten content rises from 10.21 wt% in the Ni–1W sample to 35.77 wt% in the Ni–6W sample when the Na_2_WO_4_·2H_2_O concentration was raised from 0.01 to 0.06 mol L^−1^.

#### 3.2.3 XRD study

**Fig. 3:**
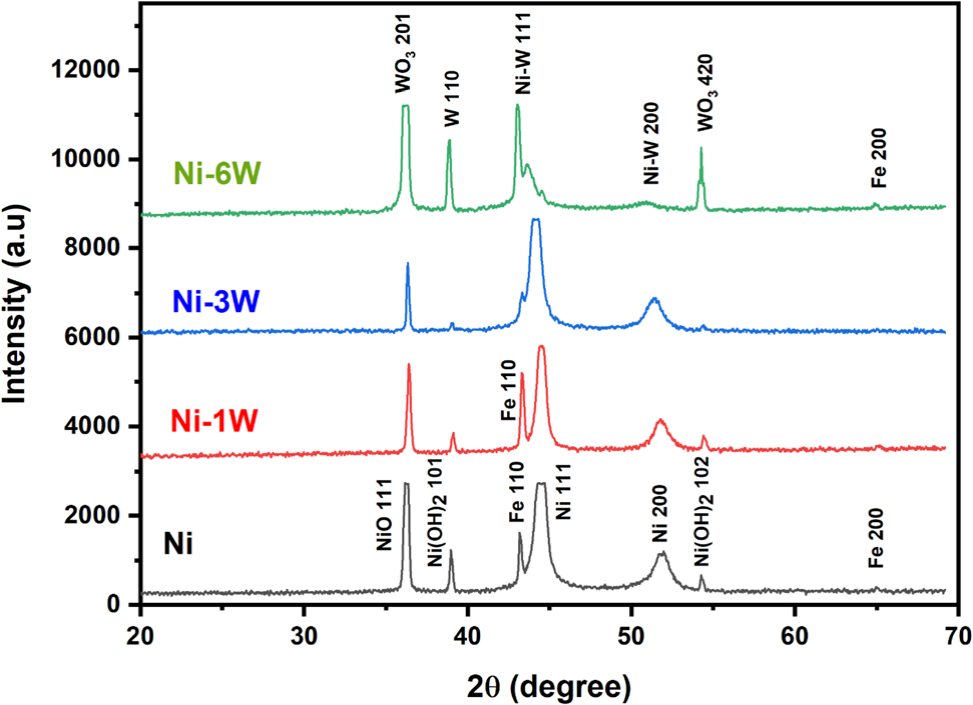
XRD patterns of electrodeposited Ni, Ni–1W, Ni–3W, and Ni–6W alloys.

XRD was used to analyze the crystal structure of the electrodeposited coatings. Fig. 3 displays the XRD spectra for Ni–1W, Ni–3W, and Ni–6W coatings. The sharp peaks in the patterns indicate the crystalline nature of all the deposits. Using Scherrer's equation, the average particle sizes were calculated to be 23.9 nm for Ni, 11.2 nm for Ni–1W, 8.3 nm for Ni–3W, and 7.3 nm for Ni–6W, showing a decrease in particle size with increasing tungsten content. The Ni coating XRD pattern revealed the presence of metallic Ni (indicated by the (200) and (111) planes, JCPDS file 45-1027), NiO (confirmed by the (111) plane, JCPDS file 78-0643), and β-Ni(OH)_2_ (identified by the (102) and (101) planes, JCPDS file 14-0117), in addition to the Fe substrate peaks (the (200) and (110) planes, JCPDS file 06-0696). The Ni–1W alloy coating's XRD pattern showed the presence of Ni_17_W_3_ (represented by the (111) and (200) planes, JCPDS file 65-4828), metallic W (the (110) plane, JCPDS file 04-0806), and WO_3_ (the (201) and (420) planes, JCPDS files 75-2187 and 43-1035), as well as the Fe substrate peak. The XRD data of the Ni–3W and Ni–6W alloy coatings indicated the same phases as those found in the Ni–1W coating.

#### 3.2.4 XPS study

XPS was utilized to investigate the elemental composition and chemical states of the as-prepared Ni, Ni–1W, Ni–3W, and Ni–6W electrocatalysts as shown in Fig. S1 (ESI).† All electrocatalysts exhibited XPS spectra for Ni 2p and O 1s, indicating the presence of metal oxides (NiO and WO_3_) on the coating surfaces. The Ni 2p_3/2_ region showed peaks corresponding to metallic Ni^0^, Ni^2+^, and a Ni^2+^ shakeup satellite peak. The W 4f_7/2_ region displayed peaks associated with W^0^, W^5+^, and W^6+^.

More specifically, the Ni 2p region showed peaks at approximately 852.8 eV and 855.8 eV (Ni 2p_3/2_) and around 861 eV (Ni 2p_3/2_ satellite), corresponding to Ni^0^, Ni^2+^, and the Ni^2+^ shakeup satellite, respectively. In the O 1s region, a peak at about 529.7 eV was attributed to structural oxygen (O^2−^), while peaks at approximately 531.6 eV and 533.1 eV were assigned to hydroxide groups and adsorbed water, respectively. The W 4f spectrum showed three peaks: one at 29 eV (W^0^ in W 4f_7/2_), another at approximately 34.9 eV (W^6+^ in W 4f_7/2_), and a third at around 33.7 eV (W^5+^ in W 4f_7/2_).^45–47^

### 3.3 Electrocatalytic HER performance of coatings

#### 3.3.1 Linear sweep voltammetry

**Fig. 4:**
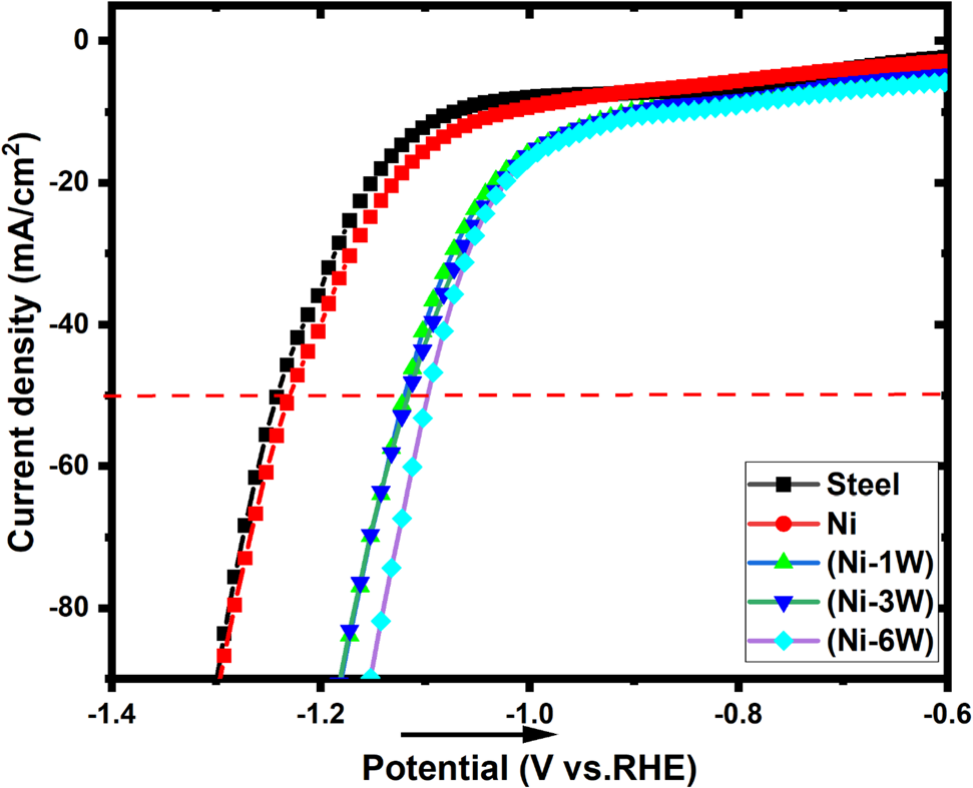
Polarization curves of the HER on the deposited electrocatalytic materials in 1 mol L^−1^ KOH at 298 K. The potential scan rate is 50 mV s^−1^.

Electrocatalytic activity for the HER was evaluated using linear sweep voltammetry in a 1 mol L^−1^ KOH solution at room temperature. The polarization curves (Fig. 4) compare the performance of the prepared electrocatalysts, a bare steel substrate, and a pure Ni coating. Higher catalytic activity is indicated by a shift of the current–potential (*I*–*E*) curve to more positive potentials, reflecting decreased cathodic polarization. Table 4 summarizes the results, including the overpotential (*η*_50_) needed for a current density of −50 mA cm^−2^. The Ni–6W coating exhibited the lowest overpotential, indicating the best performance, which is attributed to its higher tungsten content (35.77%). A lower onset potential (the potential at which the HER current significantly increases) also signifies better activity. Ni–6W composite had the most positive onset potential (−1.01 V *vs.* RHE), suggesting it initiates HER at lower applied potentials. This enhanced activity is due to the presence of tungsten. The results suggest a correlation between increased W content in the coating and improved electrocatalytic performance. Adding W to Ni enhances HER kinetics. When a water molecule adsorbs onto the Ni–W surface, the tungsten sites facilitate the formation of hydrogen gas (H_2_) from adsorbed hydrogen (H_ads_). Tungsten oxides (WO_*x*_), which may form during HER, are known to have excellent HER activity. Tungsten's hydrogen adsorption free energy is very close to that of platinum, making it an effective adsorption site for H_ads_.^37^

**Table 4:** The onset potential, the overvoltage at −50 mA cm^−2^, and the Tafel parameters of the hydrogen evolution reaction on the electrocatalytic materials under study

Sample	Onset potential (V *vs.* RHE)	*η*_50_ (V)	Tafel slope (*b*) (mV dec^−1^)	ECD (*j*_0_) (mA cm^−2^)
Steel substrate	−1.20	−0.808	−266	0.199
Ni	−1.18	−0.788	−248	0.309
Ni–1W	−1.09	−0.708	−236	0.349
Ni–3W	−1.06	−0.698	−217	0.564
Ni–6W	−1.01	−0.668	−168	0.644

#### 3.3.2 Tafel plot

Electrochemical Tafel parameters for the HER were estimated by fitting the LSV data (Fig. 4) to the Tafel equation (eqn (6)), where *η* is the overpotential, *j* is the current density, *j*_0_ is the exchange current density, and *b* is the Tafel slope.

6 *η* = *b* log(*j*/*j*_0_)

The ECD (*j*_0_) represents the reaction rate at equilibrium. In alkaline conditions, HER initiates with water molecule dissociation. The Volmer, Heyrovsky, and Tafel steps (eqn (7)–(9)) describe the possible HER steps.^10,48–54^

7 H_2_O + e^−^ + M → M–H_ads_ + OH^−^ Volmer step (*b* = −120 mV dec^−1^)

8 M–H_ads_ + H_2_O + e^−^ → M + H_2_ + OH^−^ Heyrovsky step (*b* = −40 mV dec^−1^)

9 2M–H_ads_ → 2M + H_2_ Tafel step (*b* = −30 mV dec^−1^)

**Fig. 5:**
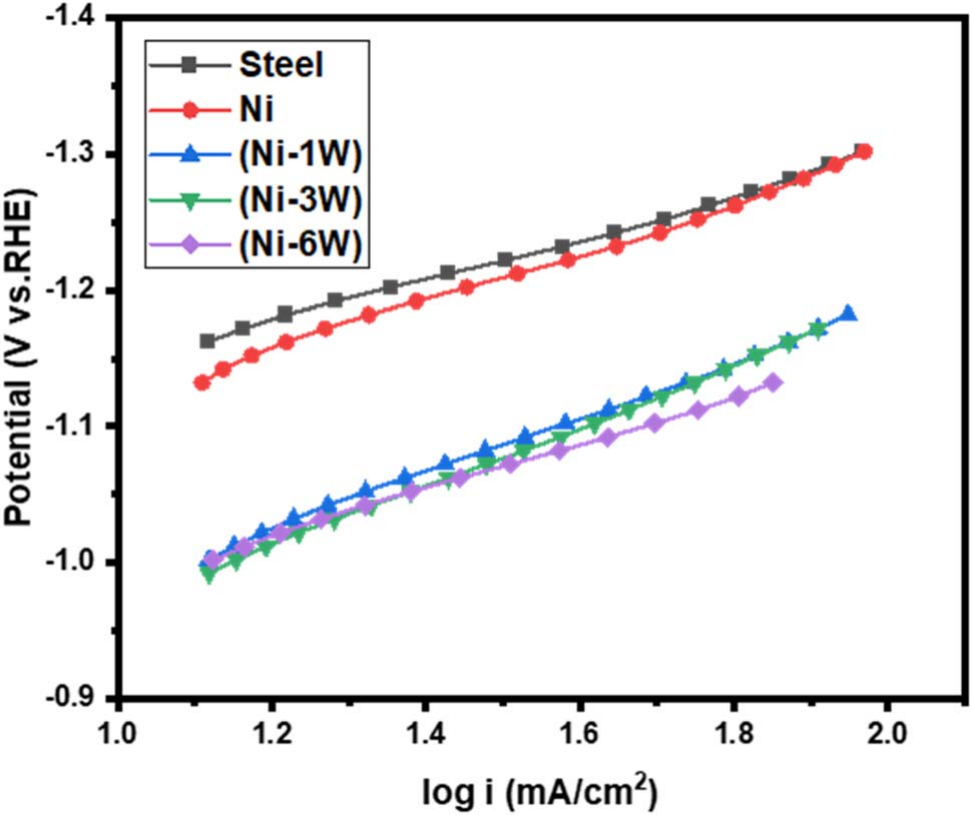
Tafel plots of hydrogen evolution reaction on the deposited electrocatalytic materials in 1 mol L^−1^ KOH at 298 K.

Steel, Ni, and Ni–1W coatings exhibited high Tafel slopes (−266 to −236 mV dec^−1^), suggesting the Volmer step (electrochemical hydrogen adsorption) may be a significant factor in the rate-determining step (RDS). The steeper slopes compared to the theoretical Volmer value (−120 mV dec^−1^) likely arise from the specific surface properties of these electrodes.^37^ Ni–3W and Ni–6W coatings showed significantly lower Tafel slopes (−217 and −168 mV dec^−1^, respectively), indicating a modification in the mechanism of reaction or a shift in the RDS with increasing tungsten content. While these lower slopes could suggest a move towards a Heyrovsky or Tafel RDS, they are not close enough to the ideal values to definitively confirm this. The Tafel slope serves as an indicator of electrocatalytic efficiency. A lower Tafel slope signifies a more rapid increase in current density with overpotential, thus a superior electrocatalyst. In this study, Ni–6W exhibited the smallest Tafel slope, suggesting it possesses the highest HER activity. Expanding the Tafel line to zero overpotential yields the ECD (*j*_0_) (Table 4). A higher *j*_0_ indicates better intrinsic catalytic activity. Ni–6W had the highest ECD (0.644 mA cm^−2^). This provides additional support for the observed superior electrocatalytic performance (Fig. 5).

#### 3.3.3 The long-term stability of the catalysts

##### 3.3.3.1 Chronopotentiometry study

**Fig. 6:**
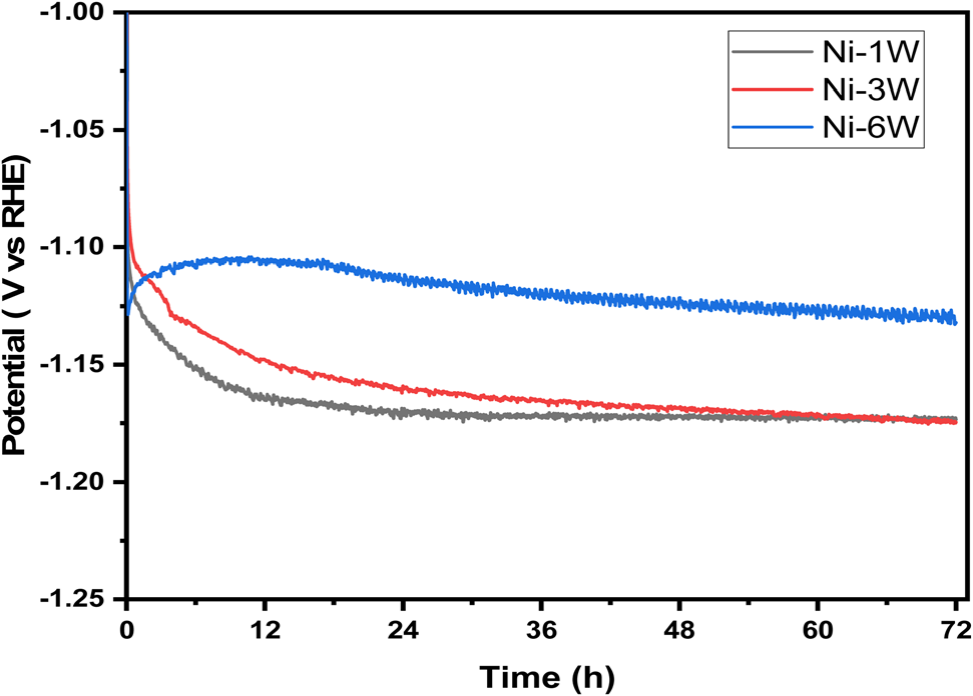
Chronopotentiometric curves for HER on the surface of Ni–1W, Ni–3W, and Ni–6W coatings at a current density of −50 mA cm^−2^.

The long-term stability of electrocatalysts is crucial for their practical use. Chronopotentiometry, a technique that measures an electrode's potential under a constant current, was used to assess this stability. Fig. 6 displays the chronopotentiometry curves for Ni–1W, Ni–3W, and Ni–6W catalysts in a 1 mol L^−1^ KOH solution at a fixed current of −50 mA cm^−2^ over 72 hours. Ni–1W, and Ni–3W samples showed a sharp initial drop in overpotential followed by a plateau.^33^ This initial drop is attributed to the rapid hydrogen ion reduction and hydrogen gas evolution immediately after the current is applied.^55^ After approximately 14 hours, the potential stabilized, indicating that equilibrium had been reached and hydrogen evolution was proceeding steadily. The observed difference in the chronopotentiometric curves, particularly at short times, for Ni–6W compared to Ni–1W and Ni–3W alloys during the HER at a constant current density of −50 mA cm^−2^ can be attributed to several factors related to the higher tungsten content in the Ni–6W alloy.^56–59^ Tungsten is known to influence the electronic properties and hydrogen binding energy of nickel. A higher tungsten content (Ni–6W) might lead to different adsorption/desorption energies for hydrogen intermediates, affecting the initial potential required to sustain the current.^58^ It is possible that Ni–6W initially has a different activation energy or requires a different initial overpotential to facilitate the initial hydrogen adsorption and subsequent reaction steps compared to Ni–1W and Ni–3W, where the Ni-rich surface might behave more like pure Ni or a low-W alloy.^58^ In addition, electrodeposited composite Ni–W alloys can exhibit varying surface morphologies and roughness depending on the tungsten content and deposition conditions. A rougher surface would mean a larger electrochemically active surface area (ECSA). At short times, a larger ECSA could lead to a more efficient distribution of the applied current, potentially resulting in a different initial potential response.^59^

Notably, the Ni–6W catalyst exhibited lower potential than the Ni–3W and Ni–1W catalysts throughout the entire 72-hour period. This demonstrates that Ni–6W is more effective for the HER at −50 mA cm^−2^, as it requires less voltage to produce the same current density, signifying higher electrocatalytic activity.

##### 3.3.3.2 LSV study

**Fig. 7:**
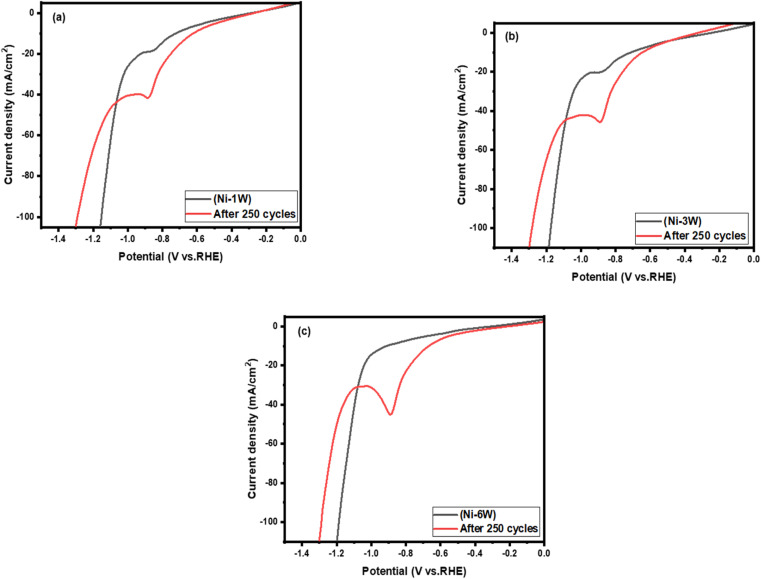
LSV for (a) Ni–1W, fresh and after 250 cycles; (b) Ni–3W, fresh and after 250 cycles; and (c) Ni–6W, fresh and after 250 cycles. The curves are collected in 1 mol L^−1^ KOH at 298 K with a scan rate of 50 mV s^−1^.

LSV is a valuable tool for assessing a material's stability in its working environment by tracking changes in the LSV profile after repeated cycles.^60^ In this study, LSV measurements were conducted in 1 mol L^−1^ KOH at room temperature with a scan rate of 50 mV s^−1^ to compare the stability of composite Ni–W alloy coatings on a steel substrate before and after 250 cycles, as illustrated in Fig. 7. The data reveals the stability and performance of the coating for HER in an alkaline medium. When composite Ni–W alloy coatings are immersed in a 1 mol L^−1^ KOH electrolyte, a nickel hydroxide layer quickly forms on their surface. The cathodic peak observed in the coatings' LSV (Fig. 7) is attributed to the reduction of Ni(OH)_2_ back to metallic nickel.^61^

The onset potential for the cycled coatings (red curves) appears to be slightly less negative (shifted to the right) compared to the fresh coatings (black curves). This suggests that the activity of the catalyst towards HER after cycling was improved. In addition, there are some changes in the shape of the curve and a change in slope after cycling. This could suggest alterations in the catalyst's surface or the reaction pathway. It can be concluded that the performance of all the coatings studied for HER in an alkaline solution increased after 250 cycles. This is evidenced by the higher current densities and a positive shift in the onset potential.^62^

#### 3.3.4 EIS study

**Fig. 8:**
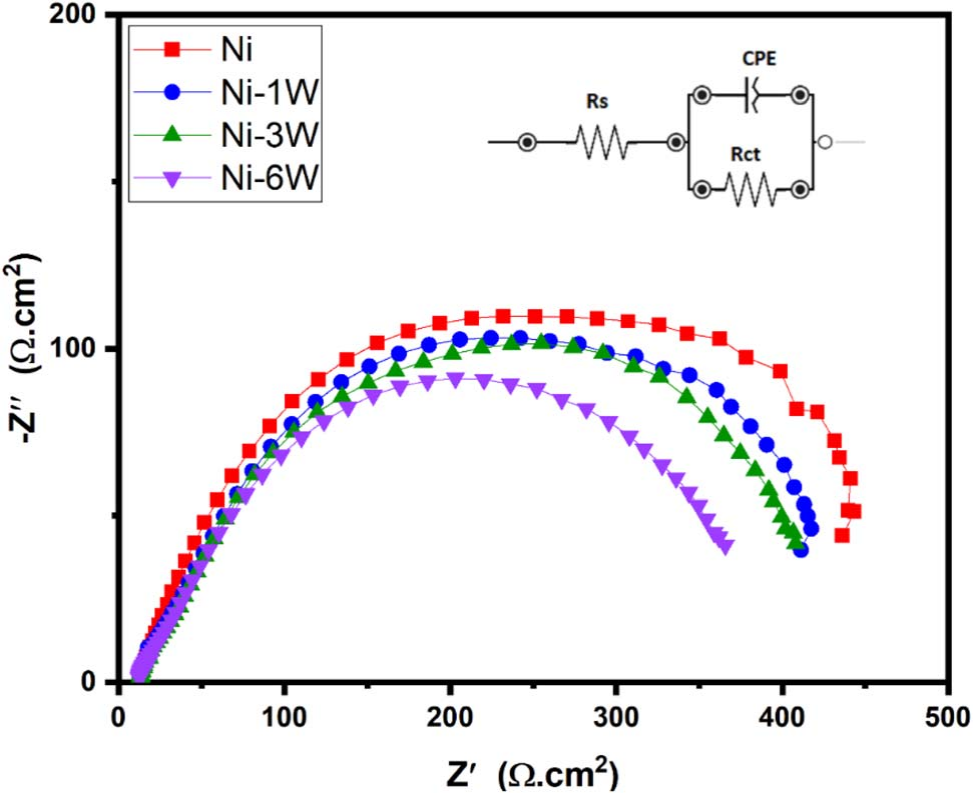
Nyquist plots for Ni, Ni–1W, Ni–3W, and Ni–6W coatings in 1 mol L^−1^ KOH at 298 K.

**Fig. 9:**
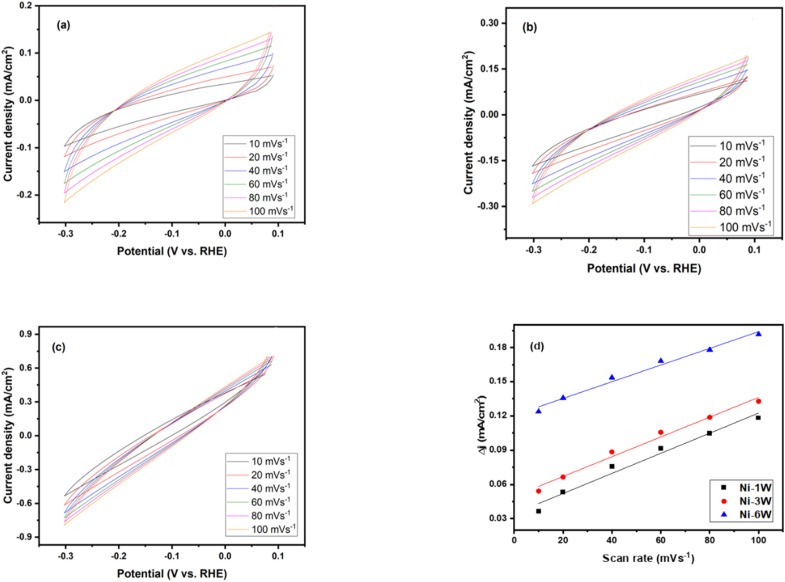
Cyclic voltammetry curves in 1 mol L^−1^ KOH at scan rates varying from 10 to 100 mV s^−1^: (a) Ni–1W, (b) Ni–3W, (c) Ni–6W. (d) The capacitive current response to varying scan rates.

To further explore the improved HER activity in alkaline conditions, EIS was performed. Fig. 8 shows Nyquist plots with a single compressed semicircle for all samples, suggesting a similar electrochemical reaction pathway. These plots were fitted using an equivalent circuit model (inset of Fig. 9), and the fitting results are summarized in Table 5. The semicircle's diameter denotes the charge transfer resistance (*R*_ct_), while *R*_s_ represents the solution resistance, and CPE (constant phase element) is associated with the double-layer capacitance (*C*_dl_). A smaller diameter indicates a lower *R*_ct_ and faster electron transfer.^63^ As shown in Fig. 9 and Table 5, the Ni–6W sample exhibits the lowest *R*_ct_ (353.23 Ω cm^2^), followed by Ni–3W (395.03 Ω cm^2^), and then Ni–1W (404.76 Ω cm^2^). This indicates that the Ni–6W alloy coating has the fastest HER kinetics and has the greatest inherent ability to facilitate the HER. The EIS data were modeled using an equivalent circuit comprising a solution resistance (*R*_s_), a charge transfer resistance (*R*_ct_), and a constant phase element (CPE) representing the interfacial capacitance. The *C*_dl_ was calculated using the following formula:^63^

10 *C*_dl_ = *Y*_0_(*ω*_max_)^*n*−1^

where *Y*_0_ is a proportionality coefficient, *ω*_max_ is the frequency at which the imaginary component (*Z*_imag_) reaches its highest value, and *n* is a parameter describing the phase shift.

**Table 5:** Electrochemical parameters estimated from EIS data for Ni, Ni–1W, Ni–3W, and Ni–6W coatings in 1 mol L^−1^ KOH at 298 K

Sample	*R*_s_ (Ω cm^2^)	*R*_ct_ (Ω cm^2^)	*C*_dl_ (F cm^−2^)
Ni	12.65	430.78	1.30 × 10^−4^
Ni–1W	12.70	404.76	1.88 × 10^−4^
Ni–3W	13.09	395.03	2.02 × 10^−4^
Ni–6W	12.43	353.23	2.59 × 10^−4^

#### 3.3.5 ECSA estimations

The electrochemical surface area (ECSA) of the Ni–1W, Ni–3W, and Ni–6W coatings was determined by analyzing the double-layer capacitance (*C*_dl_). Cyclic voltammetry was conducted in a potential range (0.1 to −0.3 V) where no faradaic reactions occur, using different scan rates (10–100 mV s^−1^) in 1 mol L^−1^ KOH (Fig. 9a–c). The difference between cathodic and anodic current densities (Δ*J*) was plotted *versus* the scan rate, and the slope of the resulting linear fit yielded the *C*_dl_ value. The ECSA was then calculated using eqn (11):11
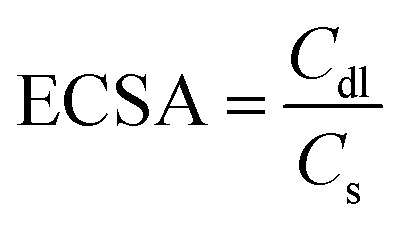
where *C*_s_ is the specific capacitance, assumed to be 40 μF cm^−2^ in this study.^64^Fig. 9(d) displays the relation between capacitive current and scan rate. The Ni–6W catalyst exhibited the highest *C*_dl_ (8.13 × 10^−4^ mF cm^−2^), followed by Ni–3W (7.57 × 10^−4^ mF cm^−2^) and Ni–1W (7.27 × 10^−4^ mF cm^−2^). This indicates that Ni–6W has the largest number of active sites. A larger ECSA generally correlates with better catalytic activity due to the increased contact area between the catalyst and reactants, leading to faster reaction rates.^63^

#### 3.3.6 XRD of electrocatalysts after stability test

**Fig. 10:**
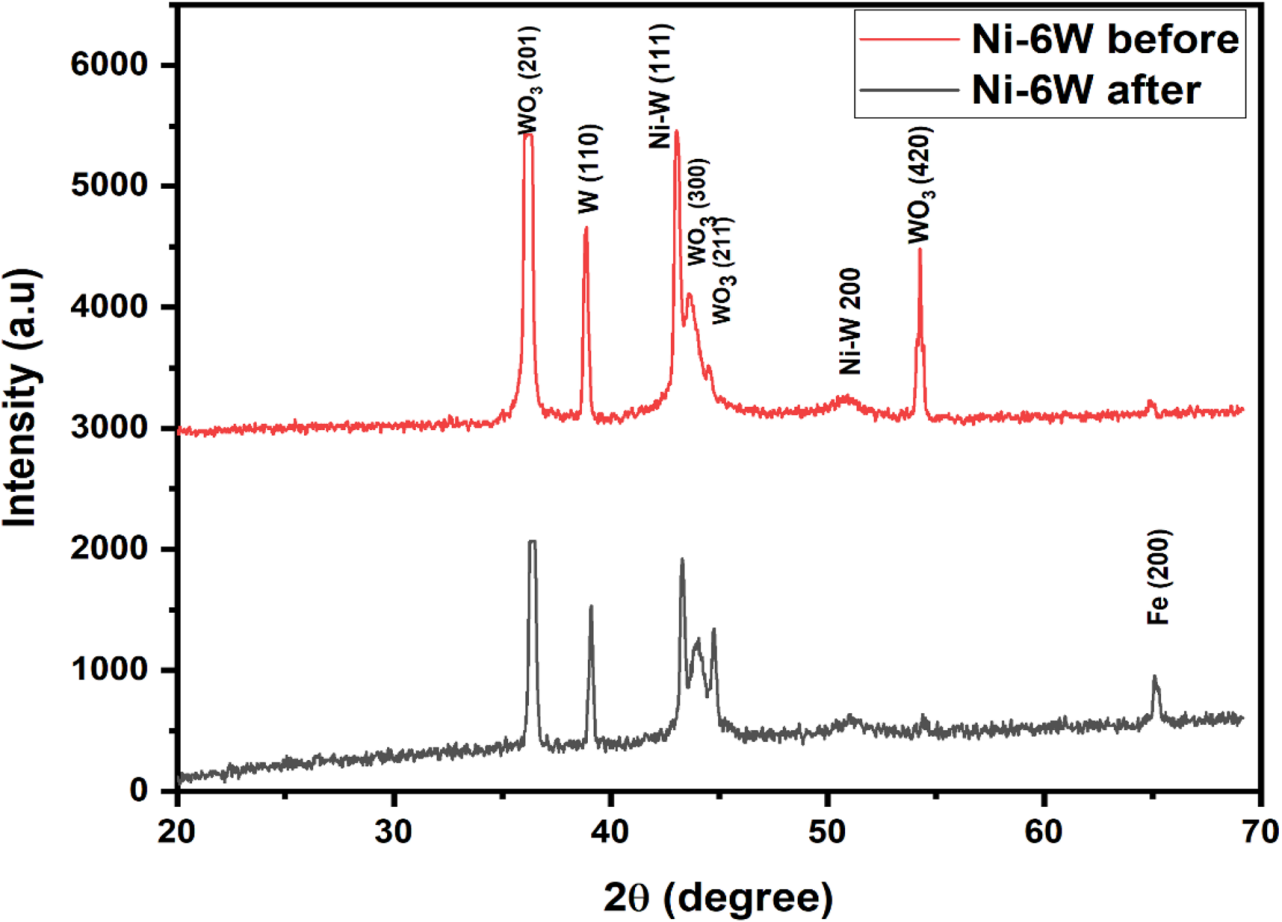
XRD patterns of electrodeposited Ni–6W alloy before and after stability test.

In this part, an analysis was conducted using XRD to show whether the composition of the best nanostructured composite Ni–W alloy (Ni–6W) changed after the stability test or not. Fig. 10 shows the XRD spectra of the Ni–6W alloy before and after the stability test. The presence of the Ni–W peaks in both patterns confirms that the main alloy structure is preserved. A noticeable decrease in the intensity of the tungsten oxide peaks (WO_3_) is observed after the stability test. This could indicate some reduction of the tungsten oxides or a change in their crystallinity. Subtle changes in the Ni–W peaks (shifts in position or changes in intensity) might suggest some changes in the Ni–W alloy structure because of the stability test. However, these changes are small. The peak labeled Fe(200) is from the steel substrate on which the Ni–6W coating is deposited. The fact that it is visible suggests that the coating might be thin or that the X-rays are penetrating through to the substrate.^63^ In conclusion, the XRD pattern suggests that the Ni–6W coating maintains its primary structure after the stability test.

#### 3.3.7 How tungsten optimizes Ni–W coatings for rapid hydrogen evolution reaction?

In electrocatalysis, it is a well-established fact that Ni–W alloys exhibit improved HER activity as their tungsten content increases.^65–67^ This enhanced hydrogen production efficiency is a result of the synergistic interplay between nickel and tungsten. When tungsten is added to nickel, it alters the electronic structure of the nickel surface, which in turn optimizes the binding energy of hydrogen intermediates (H*) on the catalyst. This is crucial for HER, as pure nickel tends to bind H* too strongly, impeding hydrogen molecule desorption, whereas pure tungsten might bind them too weakly. The resulting optimal binding energy in the alloy improves both the initial adsorption of protons and the final desorption of hydrogen gas.^65^

Increasing the tungsten content in Ni–W alloys promotes the development of amorphous or nanocrystalline structures (Fig. 1). This structural characteristic enhances the density of active sites, like grain boundaries and defects, thereby furnishing more reaction points for the HER compared to materials with a well-ordered crystalline arrangement.^66^ Higher tungsten levels in electrodeposited Ni–W alloys, particularly under specific plating conditions like high current density, commonly lead to porous or cracked surface structures (Fig. 1d for Ni–6W). This increased porosity and roughness directly expands the electrochemically active surface area (ECSA). A larger ECSA (8.13 × 10^−4^ mF cm^−2^ for Ni–6W), in turn, provides more sites for the HER, resulting in higher current densities at lower overpotentials.^67^ In addition, tungsten is known to influence the Volmer, Heyrovsky, and Tafel steps of the HER mechanism. The presence of W can lower the activation energy for these steps, accelerating the overall reaction rate.^65^ Xiong *et al.*^65^ reported that the interaction between Ni and W atoms on the surface can facilitate the transfer of electrons and protons, making the hydrogen adsorption and desorption processes more favorable.

### 3.4 A comparison of the studied electrocatalyst's performance with previously reported electrocatalysts

Table 6 compares the HER electrocatalytic performance of the Ni–1W, Ni–3W, and Ni–6W catalysts developed in this study with previously reported catalysts. The exchange current density (ECD) values demonstrate that these new catalysts exhibit superior HER activity in alkaline media compared to other materials documented in the literature. The obtained overpotential values for the present composite Ni–W alloys fall within the broad range of values reported in the literature for similar Ni–W systems, even if they are not always the lowest reported. This demonstrates that our results are valid and comparable to other studies, even if specific performance metrics vary due to differences in experimental conditions (*e.g.*, specific electrolyte concentration, temperature, deposition parameters, resulting microstructure, and exact W content). This work confirms the general feasibility and potential of composite Ni–W alloys as good electrocatalysts for HER.

**Table 6:** Comparison of electrocatalytic performance for Ni–W alloy coatings

Electrocatalyst	W content (wt%)	Media	Overpotential (*η*) (V)	Exchange current density (*i*_0_) (mA cm^−2^)	References
Ni, 99.5%, forged	—	1 M KOH	—	1.21 × 10^−4^	68
Ni	—	30 wt% NaOH	*η*_200_ = −0.586	1.5 × 10^−2^	13
Ni–W	—	1 M KOH	*η*_100_ = −0.602	2.9 × 10^−2^	37
Ni–W	5	30 wt% NaOH	*η*_200_ = −0.584	2.4 × 10^−2^	13
Ni–W	20	30 wt% NaOH	*η*_200_ = −0.503	7.3 × 10^−2^	13
Ni–W	29	30 wt% NaOH	*η*_200_ = −0.419	5.5 × 10^−1^	13
CNiW10-1	30.51	1 M NaOH	*η*_50_ = −0.341	0.012	33
PNiW5-6	14.45	1 M NaOH	*η*_50_ = −0.331	0.062	33
Ni–W	18.1	30 wt% KOH, 25 °C	—	6.5 × 10 ^−3^	69
Ni–W	10	1 M NaOH, 30 °C	—	5.0 × 10^−2^	70
Ni–23W	23	1 M KOH	*η*_100_ = −0.556	6.67 × 10^−2^	37
Ni–32W	32	1 M KOH	*η*_100_ = −0.534	10.2 × 10^−2^	37
Ni	—	1 M KOH	*η*_50_ = −0.788	0.309	This work
Ni–1W	10.2	1 M KOH	*η*_50_ = −0.708	0.349	This work
Ni–3W	16.2	1 M KOH	*η*_50_ = −0.698	0.564	This work
Ni–6W	35.8	1 M KOH	*η*_50_ = −0.668	0.644	This work

## 4. Conclusions

Ni–W nanocrystalline composite electrocatalysts were electroplated onto steel substrates from a green lactate bath containing nickel sulfate, sodium tungstate, lactic acid, sodium sulfate, and ammonium hydroxide. The resulting nanostructured composite Ni–W alloy catalysts were characterized by microstructure, morphology, composition, and HER electrocatalytic performance in 1 M KOH. Higher tungsten content led to a distinctive cracked and wrinkled surface morphology. Increasing the current density from 3.33 to 10.0 mA cm^−2^ increased the tungsten content in the alloys from 6.35 to 16.17 wt%. The average particle sizes were found to be 11.2 nm for Ni–1W, 8.3 nm for Ni–3W, and 7.3 nm for Ni–6W. XRD analysis of the composite Ni–W alloy coatings revealed the presence of Ni_17_W_3_, metallic W, and WO_3_. The Ni–6W coating maintained its primary structure after a stability test for 72 hours. XPS analysis confirmed the presence of tungsten oxide (WO_3_) on the catalyst surfaces. The Ni–6W catalyst demonstrated the best HER electrocatalytic activity, exhibiting the lowest Tafel slope (−168 mV dec^−1^) and the highest exchange current density (0.644 mA cm^−2^). LSV showed the Ni–6W coating's remarkable stability for HER in alkaline solution, retaining good activity after 250 cycles. Chronopotentiometry confirmed the superior HER performance of Ni–6W at −50 mA cm^−2^. EIS results also suggested that the Ni–6W coating is a good catalyst for HER in 1 M KOH. Tafel slope analysis suggested that the Volmer reaction might be a significant factor in the rate-determining step for the Ni–1W coating. With increasing tungsten content, the Tafel slopes decreased for Ni–3W and Ni–6W, suggesting a modification in the reaction mechanism or rate-determining step. ECSA studies, based on double-layer capacitance (*C*_dl_), showed that Ni–6W had the highest *C*_dl_ value, followed by Ni–3W and Ni–1W. Overall, these new Ni–W catalysts demonstrate good HER activity in alkaline media.

## Author contributions

R. Alsaiari: performed the spectroscopic studies, formal analysis, writing, and review of the article. A. Abd-Ellah: fabrication and characterization of catalysts, formal analysis, writing, the technical, and discussion of the article. Z. Anwar: formal analysis, coordination, review & editing of the article. S. Shata: XRD analysis, formal analysis, writing, and review of the article. M. Kamel: software generation, formal analysis, writing, and review of the article. N. Mostafa: software generation, formal analysis, writing and review of the article. The manuscript was written through contributions from all authors. All authors have given approval to the final version of the manuscript.

## Conflicts of interest

There are no conflicts to declare.

## Supplementary Material

RA-015-D5RA03136B-s001

## Data Availability

The authors confirm that the data supporting the findings of this study are available within the article and its ESI.†
