# Supplementary material for: Electrodeposition and characterization of nanostructured composite Ni–W alloys for hydrogen evolution in basic media
Source: RSC Adv. 2025 Jul 1;15(28):22322–35. doi: 10.1039/d5ra03136b (PMC12210228; doi:10.1039/d5ra03136b)
Supplement: RA-015-D5RA03136B-s001 [file RA-015-D5RA03136B-s001.pdf]

Supplementary Information (SI) for RSC Advances.
This journal is © The Royal Society of Chemistry 2025

Supplementary information

Electrodeposition and characterization of nanostructured composite Ni-W alloys for hydrogen evolution in basic media

Raiedhah A. Alsaiari[a], Alaa A. Abd- Ellah[b], Zeinab M. Anwar[b], Salah S. Shata[c], Medhat M. Kamel [b], Nasser Y. Mostafa[b*]

[a] Empty Quarter Research Centre, Department of Chemistry, Faculty of Science and Arts in Sharurah, Najran University, Sharurah, Saudi Arabia.

[b*]Department of Chemistry, Faculty of Science, Suez Canal University, Ismailia, 41522, Egypt.

[c] Geology Department, Faculty of Science, Suez Canal University, Ismailia, 41522, Egypt.

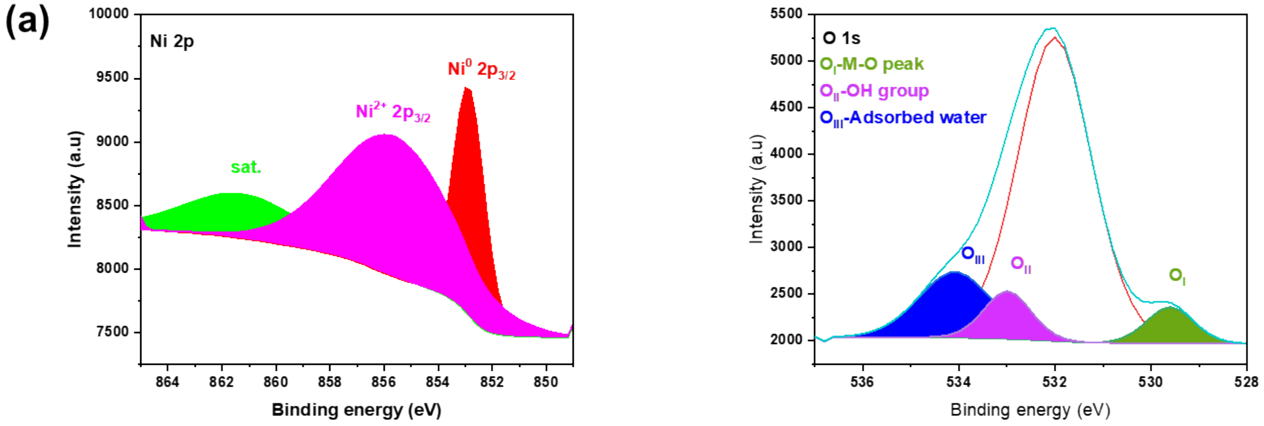

(b)

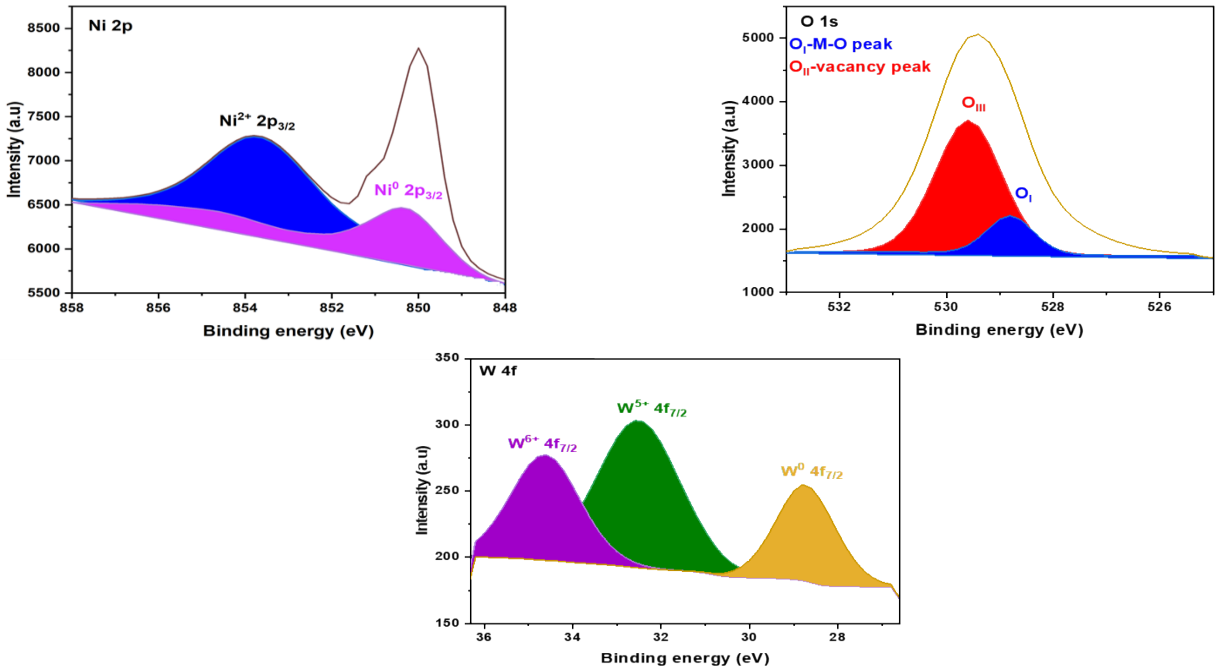
Ni 2p
Ni²⁺ 2p3/2
Ni⁰ 2p3/2
Intensity (a.u)
Binding energy (eV)
O 1s
OI-M-O peak
OII-vacancy peak
OIII
OI
Intensity (a.u)
Binding energy (eV)
W 4f
W⁶⁺ 4f7/2
W⁵⁺ 4f7/2
W⁰ 4f7/2
Intensity (a.u)
Binding energy (eV)

(c)

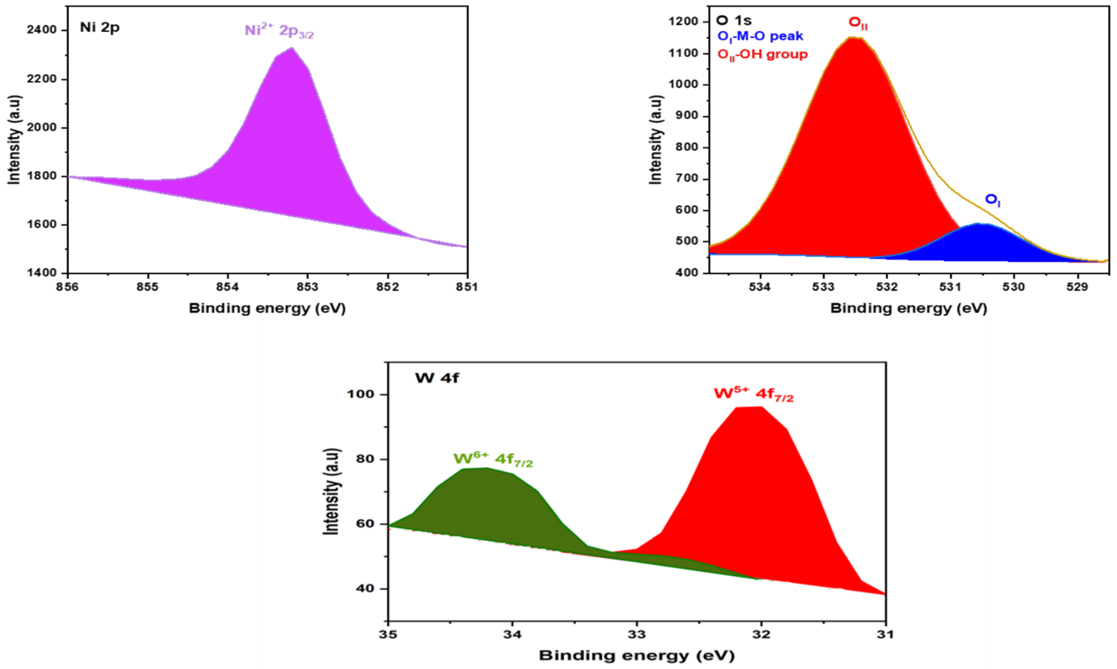
Ni 2p
Ni²⁺ 2p3/2
Intensity (a.u)
Binding energy (eV)
O 1s
OI-M-O peak
OII-OH group
OII
OI
Intensity (a.u)
Binding energy (eV)
W 4f
W⁶⁺ 4f7/2
W⁵⁺ 4f7/2
Intensity (a.u)
Binding energy (eV)

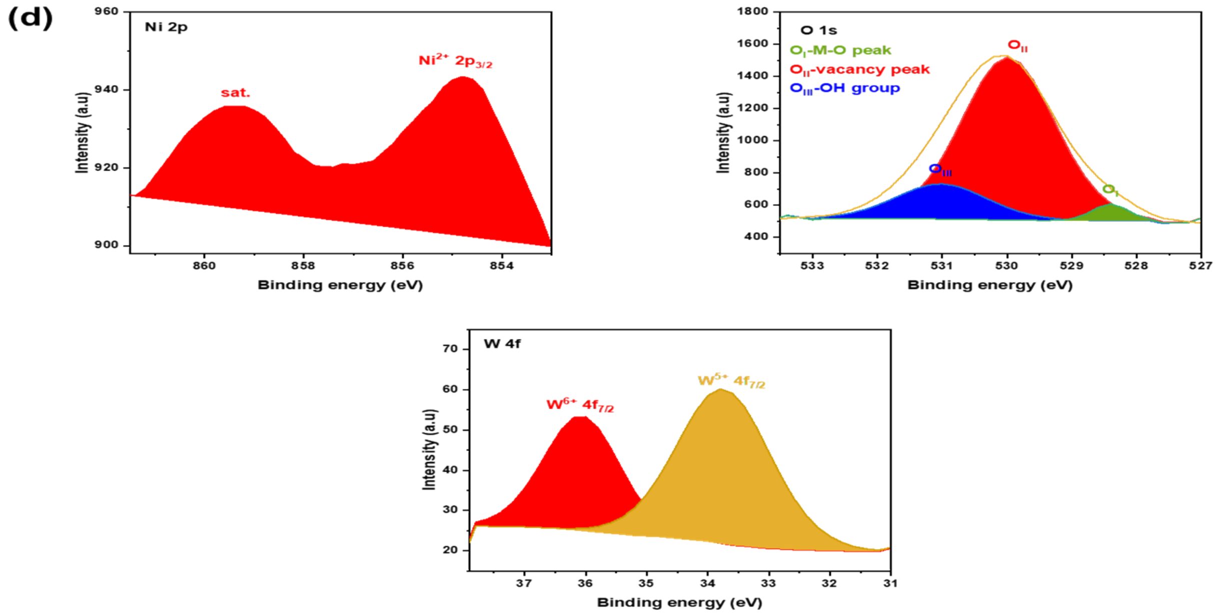

Fig. S1: XPS of electrodeposited Ni (a), Ni-1W (b), Ni-3W (c) and Ni-6W (d) alloys
